# A Kinome-Wide Synthetic Lethal CRISPR/Cas9 Screen Reveals That mTOR Inhibition Prevents Adaptive Resistance to CDK4/CDK6 Blockade in HNSCC

**DOI:** 10.1158/2767-9764.CRC-24-0247

**Published:** 2024-07-29

**Authors:** Yusuke Goto, Keiichi Koshizuka, Toshinori Ando, Hiroki Izumi, Xingyu Wu, Kuniaki Sato, Tomohiko Ishikawa, Kyle Ford, Xiaodong Feng, Zhiyong Wang, Nadia Arang, Michael M. Allevato, Ayush Kishore, Prashant Mali, J. Silvio Gutkind

**Affiliations:** 1 Moores Cancer Center, University of California San Diego, La Jolla, California.; 2 Graduate School of Biomedical and Health Sciences, Hiroshima University, Hiroshima, Japan.; 3 Department of Bioengineering, University of California San Diego, San Diego, California.

## Abstract

The comprehensive genomic analysis of the head and neck squamous cell carcinoma (HNSCC) oncogenome revealed the frequent loss of p16^INK4A^ (*CDKN2A*) and amplification of cyclin D1 genes in most human papillomavirus–negative HNSCC lesions. However, cyclin-dependent kinase 4 and 6 (CDK4/6) inhibitors have shown modest effects in the clinic. The aberrant activation of the PI3K/mTOR pathway is highly prevalent in HNSCC, and recent clinical trials have shown promising clinical efficacy of mTOR inhibitors (mTORi) in the neoadjuvant and adjuvant settings but not in patients with advanced HNSCC. By implementing a kinome-wide CRISPR/Cas9 screen, we identified cell-cycle inhibition as a synthetic lethal target for mTORis. A combination of mTORi and palbociclib, a CDK4/6-specific inhibitor, showed strong synergism in HNSCC-derived cells *in vitro* and *in vivo*. Remarkably, we found that an adaptive increase in cyclin E1 (CCNE1) expression upon palbociclib treatment underlies the rapid acquired resistance to this CDK4/6 inhibitor. Mechanistically, mTORi inhibits the formation of eIF4G–*CCNE1* mRNA complexes, with the consequent reduction in mRNA translation and CCNE1 protein expression. Our findings suggest that mTORi reverts the adaptive resistance to palbociclib. This provides a multimodal therapeutic option for HNSCC by cotargeting mTOR and CDK4/6, which in turn may halt the emergence of palbociclib resistance.

**Significance:**

A kinome-wide CRISPR/Cas9 screen identified cell-cycle inhibition as a synthetic lethal target of mTORis. A combination of mTORi and palbociclib, a CDK4/6-specific inhibitor, showed strong synergistic effects in HNSCC. Mechanistically, mTORis inhibited palbociclib-induced increase in CCNE1.

## Introduction

Head and neck squamous cell carcinoma (HNSCC) is among the 10 most frequent cancers in the United States, with 54,540 new cases and 11,580 deaths estimated in the United States alone in 2023 (1). Recent breakthrough treatment options by the use of immunotherapies targeting immune checkpoints brought survival benefit for patients with HNSCC; however, the overall response rate to these immunotherapies in HNSCC is only ∼20% (2). Thus, novel therapeutic options for this disease are urgently needed.

The comprehensive analysis of the HNSCC oncogenome revealed frequent loss of p16^INK4A^ (*CDKN2A*) in human papillomavirus–negative (HPV^−^) HNSCC, which accounts for 60% of the cases (3). Furthermore, amplification of the cyclin D1 (*CCND1*) gene is a frequent event in HNSCC, which has been reported to include 31% of the HPV^−^ HNSCC (3). However, cyclin-dependent kinase 4 and 6 (CDK4/6) inhibitors as single agents have shown modest effects regardless of *CDKN2A-*altered status in recurrent and metastatic HNSCC (4). Furthermore, a double-blind, randomized phase II trial (PALATINUS) that evaluated the efficacy of palbociclib plus cetuximab in patients with unselected HPV-unrelated recurrent or metastatic HNSCC did not significantly prolong the overall survival (OS) of patients with HNSCC (5). In this context, novel combinatory therapeutics for palbociclib based on molecular biological mechanisms are needed. On the other hand, our group has been focusing on the study of mTOR signaling in HNSCC. Indeed, we have shown that the PI3K–mTOR pathway is the most frequently activated signaling mechanism in HNSCC, as judged by strong pS6 expression in more than 90% of HNSCC specimens (6). Based on these results, we have recently performed a clinical trial using rapamycin, which is a first-generation mTOR inhibitor (mTORi), in newly diagnosed patients with HNSCC. Here, we found that rapamycin was effective for most of the patients with HNSCC, with an overall response rate of 25% including one case of complete response despite 21-day treatment duration (7). Similarly, we have recently shown that mTOR inhibition with everolimus significantly diminishes the progression-free survival of locally advanced HPV^−^ HNSCC lesions in the adjuvant setting (8). However, earlier clinical trials involving patients with advanced, recurrent metastatic HNSCC showed limited response and resulted in treatment failure (9). The molecular mechanisms underlying mTORi resistance should be uncovered to find precise molecular targets which can be combined with mTORis to achieve durable responses.

In this study, we aimed to identify synthetic lethal targets and resistance mechanisms for mTORis by taking advantage of CRISPR/Cas9 screening. Using a second-generation mTORi, INK128, we identified the cell-cycle regulation pathway as one of the most significant synthetic lethal targets and resistant pathways to mTORis in HNSCC. To explore the translational potential of these findings, we observed a strong synergism between INK128 and palbociclib, which is a widely used approved CDK4/6 inhibitor, *in vitro* and *in vivo*. In turn, we found that CCND1 and cyclin E1 (CCNE1) accumulate upon palbociclib treatment and that CCNE1 overexpression is sufficient to induce palbociclib resistance in HNSCC cells. Coadministration of INK128 and palbociclib could prevent the protein accumulation of CCNE1 by reducing its mRNA translation, and consequently, coadministration of these targeted agents can revert the resistance to palbociclib in CCNE1-overexpressing cells. Overall, our findings suggest that cotargeting mTOR and cell-cycle signaling represents a potential therapeutic option for HNSCC. These findings may be also relevant for other cancer types characterized by the progressive acquisition of resistance to CDK4/6 inhibitors.

## Materials and Methods

### Cell lines, culture conditions, and chemicals

Human HNSCC cell lines Cal27 (RRID: CVCL_1107) and HN12 (RRID: CVCL_HN12) were genetically characterized as part of NIH/National Institute of Dental and Craniofacial Research (NIDCR) Oral and Pharyngeal Cancer Branch cell collection given from NIH/NIDCR in 2016 and have been described previously (10). Only cell lines of <20 passages were used for experiments. All cell lines were frequently tested for *Mycoplasma* contamination. No presence of *Mycoplasma* was found according to MycoAlert (#LT07-418, Lonza, ME, USA). All cell lines were cultured in DMEM (D-6429, Sigma-Aldrich, St. Louis, MO), 10% FBS (F2442, Sigma-Aldrich), and 1% antibiotic/antimycotic solution (A5955, Sigma-Aldrich), under 5% CO_2_, at 37°C. INK128 (I-3344) was purchased from LC Laboratories (Woburn, MA), and palbociclib (S1116) was purchased from Selleckchem (Houston, TX).

### CRISPR screen

CRISPR screen was performed as previously described (11–13). The two-vector system was used for this study. First, we generated Cas9–stably expressing Cal27 cells with lentiviral infection from lentiCas9-Blast. lentiCas9-Blast was a gift from Feng Zhang (Addgene plasmid # 52962; http://n2t.net/addgene:52962; RRID: Addgene_52962). The infected cells were selected with blasticidin (10 µg/mL) for 10 days. After confirming Cas9 expression by Western blot, the Cal27-Cas9 cells were infected with two different guide RNAs targeting the AAVS locus and were subjected to next-generation sequencing (NGS) to confirm cutting efficiency. The CRISPR cutting efficiency of Cas9-expressed cells was tested by two AAVS locus–targeting single-guide RNAs (sgRNA): gT1 and gT2 (hygromycin B resistant). The *AAVS1* gene from genome DNA was amplified, and then NEBNext primers (E7335S); New England Biolabs, Ipswich, Massachusetts were used to attach the sequencing adapters. Sequencing data were analyzed using CRISPResso2 (http://crispresso.rocks/; ref. 14). AAVS1 locus–targeting sgRNA constructs (gT1/gT2) were provided by Prashant’s laboratory. Primer sequences for NGS are as follows: NGS_AAVS1_F: ACA​CTC​TTT​CCC​TAC​ACG​ACG​CTC​TTC​CGA​TCT​TCC​CAG​GGC​CGG​TTA​ATG​TGG; NGS_AAVS1_R: GAC​TGG​AGT​TCA​GAC​GTG​TGC​TCT​TCC​GAT​CTT​GCC​TAA​CAG​GAG​GTG​GGG​GTT​AG; amplicon reference sequence (±30 bp around the PAM seq): TGC​CTA​ACA​GGA​GGT​GGG​GGT​TAG​ACC​CAA​TAT​CAG​GAG​ACT​AGG​AAG​GAG​GAG​GCC​TAA​GGA​TGG​GGC​TTT​TCT​GTC​ACC​AAT​CCT​GTC​CCT​AGT​GGC​CCC​ACT​GTG​GGG​TGG​AGG​GG.

Next, Cal27-Cas9 cells were infected with a human kinome CRISPR pooled library (Brunello, RRID: Addgene_75312) at representation of 650 and a multiplicity of infection of 0.3. The viral titer of lentivirus was analyzed using the qRT-PCR titer kit (#631235, Takara, Mountain View, CA) and functional titration. The human kinome CRISPR pooled library (Brunello) was a gift from John Doench and David Root (RRID: Addgene_75312). Cal27-Cas9-kinome library cells were treated with two different groups: vehicle-treated or INK128 (10 nmol/L)-treated group, with triplicate. For PD 0 and PD 20 samples, the barcode was PCR-recovered from genomic samples, and samples were sequenced to calculate the abundance of the different sgRNA probes. PCR of sgRNA for Illumina sequencing protocol was obtained from the Broad Institute (https://portals.broadinstitute.org/gpp/public/resources/protocols). The change in the relative abundance of each sgRNA in the library over time is measured using PinAPL-Py software (15). Significantly changed hit sgRNAs were extracted with adjusted *P* value < 0.001. The hit sgRNAs were subjected to pathway analysis using Enrichr software (16). The Kyoto Encyclopedia of Genes and Genomes (KEGG) pathway combined score was calculated with a *P* value and *z*-score using the formula *c* = log (*p*) × *z*, in which *c* is the combined score, *p* is the Fisher exact test *P* value, and *z* is the *z*-score (17). NGS was conducted by Institute for Genomic Medicine Genomics Center in UC San Diego. Sequencing data from the CRISPR screen were deposited into Sequence Read Archive (BioProject ID: PRJNA1119544).

### Antibodies

Antibodies against CCND1 (Cat# 2978, RRID: AB_2259616), CCNE1 (Cat# 20808, RRID: AB_2783554), pRb (Cat# 9307, RRID: AB_330015), Rb (Cat# 9309, RRID: AB_823629), pS6 (Cat# 2211, RRID: AB_331679), S6 (Cat# 2217, RRID: AB_331355), pERK (Cat# 4370, RRID: AB_2315112), ERK (Cat# 9102, RRID: AB_330744), pAKT (Cat# 4060, RRID: AB_2315049), AKT (Cat# 9272, RRID: AB_329827), CDK4 (Cat# 12790, RRID: AB_2631166), eIF4E (Cat# 9742, RRID: AB_823488), Cas9 (Cat# 14697, RRID: AB_2750916), HA-Tag (Cat# 3724, RRID: AB_1549585), vinculin (Cat# 13901, RRID: AB_2728768), β-actin (Cat# 4967, RRID: AB_330288), and GAPDH (Cat# 2118, RRID: AB_561053) were purchased from Cell Signaling Technology (Beverly, MA, USA). Antibodies against eIF4G (Cat# sc-133155, RRID: AB_2095748) and CDK6 (Cat# sc-177-G, RRID: AB_631226) were purchased from Santa Cruz Biotechnology (Dallas, TX, USA). Antibodies against CCND1 (Cat# 26939-1-AP, RRID: AB_2880691) and CCNE1 (Cat# 11554-1-AP, RRID: AB_2071066) were purchased from Proteintech (Rosemont, IL, USA). Antibody against BrdU (Cat# OBT0030S, RRID: AB_609570) was purchased from Bio-Rad (Hercules, CA, USA).

### DNA constructs and viral infection

pBABE puro CCND1 HA was a gift from William Hahn (Addgene plasmid # 9050; RRID: Addgene_9050). pInducer20 CCNE1 was a gift from Jean Cook (Addgene plasmid # 109348; RRID: Addgene_109348). Plasmids were packaged into retrovirus and lentivirus in HEK293T cells, respectively, and cells were infected with viruses for 2 days. The infected cells were selected with puromycin (1 μg/mL) for 3 days or selected with G418 (1,000 μg/mL) for 7 days, respectively. To overproduce CCNE1, cells were treated with 1 μg/mL doxycycline for at least 48 hours.

### siRNA and transfection

SMARTpool si-CDK4 (#L-003238-00-0005) and si-CDK6 (#L-003240-00-0005) were purchased from Dharmacon. siRNA Universal Negative Control #1 (#SIC-001) was purchased from Sigma-Aldrich. All cells were transfected using Lipofectamine RNAiMAX Reagent (#13778075, Invitrogen) and OPTI-MEM (#31985062, Gibco) according to the manufacturer’s instructions. siRNA transfection was performed as previously described (13, 18).

### Cell viability assay

Three thousand cells were seeded in 96-well plates and treated as indicated after they attach to the plates. After treatment for 72 hours, the culture medium was supplemented with 1/100 of the culture volume of AquaBluer reagent (#6015, MultiTarget Pharmaceuticals LLC, Colorado Springs, CO, USA) for 1 to 4 hours. Absorbances at 570 nm were recorded in a BioTek Synergy Neo microplate reader.

### Synergy determination using the Chou–Talalay method and the Bliss delta score

The Chou–Talalay method was used to determine possible synergistic effects of selected drug combinations (19). Three thousand cells per well were seeded in 96-well plates. Cells were treated with either single inhibitors or combinations thereof using seven different dilutions of each inhibitor and in technical triplicate. Cell viability was measured, after 72-hour treatment, with the AquaBluer cell viability reagent (#6015, MultiTarget Pharmaceuticals LLC) with Tecan Spark multimode microplate reader (RRID: SCR_021897). Combination index (CI) values showing either synergy (<1) or antagonism (>1) were calculated using the Chou–Talalay method. The Bliss independence model assumes a stochastic process in which two drugs elicit their effects independently, and the expected combination effect was calculated using the equation IAB = IA + IB − IA × IB, in which IA and IB are the single-agent inhibition levels at fixed concentrations (20). If the experimentally measured effect of the drug combination was equal to, higher than, or lower than the expected effect (IAB), the combination was additive (Δ Bliss = 0), synergistic (<0), or antagonistic (>0), respectively.

### Orosphere assay

Cells were seeded in 24-well ultralow attachment culture plates (Corning, Corning, NY) at 500 cells per well. The medium consisted of DMEM/F12 GlutaMAX supplement medium (#10565042, Thermo Fisher Scientific), basic fibroblast growth factor (20 ng/mL, #13256029, Thermo Fisher Scientific), epithelial growth factor ( 20 ng/mL, #PHG0313, Thermo Fisher Scientific), B-27 (1:50 dilution, #17504044, Thermo Fisher Scientific), and N2 supplement (1:100 dilution, #17502-048, Thermo Fisher Scientific). Vehicle, INK128 (20 nmol/L), and palbociclib (0.4 µmol/L) were added when cells were seeded. Around 10 days after seeding, photographs were obtained, and the sizes of sphere colonies on each well were counted using a microscope.

### Colony formation assay

Thousand cells per well were seeded in 12-well plates, and vehicle, INK128 (20 nmol/L), and/or palbociclib (0.4 µmol/L) were added after they were attached. For 8to 10 days of treatment, the medium was changed every 2 to 3 days. Cell culture plates containing colonies were gently washed with PBS twice and fixed for 5 minutes with methanol/acetic acid solution (3:1) and stained for 15 minutes with 0.5% crystal violet solution diluted in methanol. Excess stain was removed by washing repeatedly with PBS. The colony area percentage was calculated using ImageJ.

### RNA isolation from HNSCC cells and quantitative PCR

HNSCC cells were treated with INK128 (30 nmol/L) and/or palbociclib (0.6 µmol/L) for 24 hours. RNA was extracted from cells using the RNeasy Plus kit (#74134, QIAGEN). Total RNA was converted to cDNA using the SuperScript VILO cDNA Synthesis kit (#11754250, Thermo Fisher Scientific). qPCR was performed using PowerUp SYBR Green Master Mix (#A25742, Thermo Fisher Scientific). mRNA levels were normalized by RPS18 expression. The following primers were used for qPCR: CCND1 fwd 5′-AGC​TGT​GCA​TCT​ACA​CCG​AC and CCND1 rev 5′-GAA​ATC​GTG​CGG​GGT​CAT​TG; CCNE1 fwd 5′-CCA​TCA​TGC​CGA​GGG​AGC and CCNE1 rev5′-GGTCACGTTTGCCTTCCTCT; RPS18 fwd 5′-AGT​CCC​TGC​CCT​TTG​TAC​ACA and RPS18 rev 5′-CGA​TCC​GAG​GGC​CTC​ACT​A.

### RNA immunoprecipitation assay

HNSCC cells were treated with INK128 (30 nmol/L) and/or palbociclib (0.6 µmol/L) for 24 hours, and the cell lysates were collected. The RNA immunoprecipitation (RIP) assay was performed using EZ-Magna RIP RNA-binding protein immunoprecipitation kit (Sigma-Aldrich, #17-701) following the manufacturer’s instructions. Antibody against eIF4G (Santa Cruz Biotechnology Cat# sc-133155, RRID: AB_2095748) was used for the part of immunoprecipitation, and mouse IgG antibody (Cell Signaling Technology Cat# 5415, RRID: AB_10829607) was used for isotype control. cDNA synthesis of input RNA and eIF4G-binding RNA was followed by qPCR.

### Western blotting

Exponentially growing cells were washed in cold PBS, lysed on ice in lysis buffer (50 mmol/L Tris-HCl, 150 mmol/L NaCl, 1 mmol/L EDTA, and 1% NP-40), supplemented with Halt Protease and Phosphatase Inhibitor Cocktail (#78440, Thermo Fisher Scientific). Cell extracts were collected, sonicated, and centrifuged to remove the cellular debris. Supernatants containing the solubilized proteins were quantified using the detergent-compatible DC protein assay kit (#5000111, Bio-Rad, Hercules, CA, USA). Equal amounts of protein were separated by SDS-PAGE and transferred to polyvinylidene difluoride membranes. For immunodetection, the membranes were blocked for 20 minutes at room temperature in 5% nonfat dry milk in TBST buffer, followed by 2 hours of incubation with the appropriate antibodies, in 3% BSA-T-TBS buffer. Detection was conducted by incubating the membranes with the horseradish peroxidase–conjugated goat anti–rabbit IgG secondary antibody (SouthernBiotech, Birmingham, AL, USA) at a dilution of 1:20,000 in 5% milk-T-TBS buffer, at room temperature for 40 minutes, and visualized with Immobilon Western Chemiluminescent HRP Substrate (EMD Millipore, Burlington, MA, USA).

### Immunoprecipitation

HNSCC cells were treated with INK128 (30 nmol/L) for 24 hours, and the cell lysates were collected. Immunoprecipitation was performed using Pierce Classic Magnetic IP/Co-IP kit (Thermo Fisher Scientific, #88804) following the manufacturer’s instructions. The input proteins and immunoprecipitation (IP) products were analyzed for indicated proteins by Western blotting.

### Animal work

All the mice studies were approved by the Institutional Animal Care and Use Committee, University of California, San Diego (protocol #S15195). To establish tumor xenografts, 2.0 × 10^6^ cells were transplanted into the flanks of athymic nude mice (female, 4–6 weeks old; Charles River Laboratories, Wilmington, MA), and when the tumor volume reached approximately 200 mm^3^, the mice were randomized into groups and treated by i.p. injection with INK128 (1 mg/kg/day, five times a week), or oral gavage with palbociclib (50 mg/kg/day, five times a week), or control diluent (10 tumors per each group). Tumor volume was calculated by using the formula length × width × width/2. The mice were euthanized at the indicated time points, and tumors were isolated for histologic and IHC evaluation.

### IHC staining

All samples were fixed in zinc formalin (Z-Fix) and embedded in paraffin; 5-μm sections were stained with hematoxylin–eosin for diagnostic purposes. For IHC studies, samples were deparaffinized and hydrated with graded ethanol, and the endogenous peroxidase was blocked with 3% H_2_O_2_ in 70% ethanol. After washing with distilled water, antigen retrieval was performed with IHC antigen retrieval solution (#00-4955-58, Invitrogen) in a microwave at the high setting. Slides were then washed with water and PBS and incubated with the primary and secondary antibodies and developed with the Elite ABC kit (Vector Laboratories, #PK-6100) and the ImmPACT DAB substrate kit (Vector Laboratories, #SK-4105). The following antibodies were used: BrdU (Bio-Rad Cat# OBT0030S, RRID: AB_609570, 1:50), pS6 (Cell Signaling Technology Cat# 2211, RRID: AB_331679, 1:300), p4EBP1 (Cell Signaling Technology Cat# 2855, RRID: AB_560835, 1:800), CCND1 (Proteintech Cat# 26939-1-AP, RRID: AB_2880691, 1:800), and CCNE1 (Proteintech Cat# 11554-1-AP, RRID: AB_2071066, 1:400). Samples were scanned using the Aperio AT2 microscope slide scanner (Leica) and analyzed using QuPath software.

### Genomic data analysis

mRNA and RPPA expression analyses were performed using publicly available data generated by The Cancer Genome Atlas consortium, accessed through cBioPortal (www.cbioportal.org; refs. 21, 22).

### Statistical analysis

All data analyses were performed using GraphPad Prism9 for macOS (GraphPad software, San Diego, CA, USA). Comparisons between experimental groups were made using one-way ANOVA with the Tukey *post hoc* test or two-way ANOVA with the Tukey *post hoc* test. OS curves were plotted according to the Kaplan–Meier method and compared using the log-rank test. Asterisks denote statistical significance (nonsignificant, *P* > 0.05; ^∗^, *P* < 0.05; ^∗∗^, *P* < 0.01; ^∗∗∗^, *P* < 0.001; ^∗∗∗∗^, *P* < 0.0001). All data are reported as mean ± SEM with at least two biologically independent replicates. The detailed statistic for each plot was described in figure legends.

### Data availability

The CRISPR screening data generated in this study are publicly available in Sequence Read Archive at BioProject ID: PRJNA1119544.

## Results

### CRISPR/Cas9 screening identifies cell-cycle regulation as a synthetic lethal mechanism for mTORis in HNSCC

**Figure 1:**
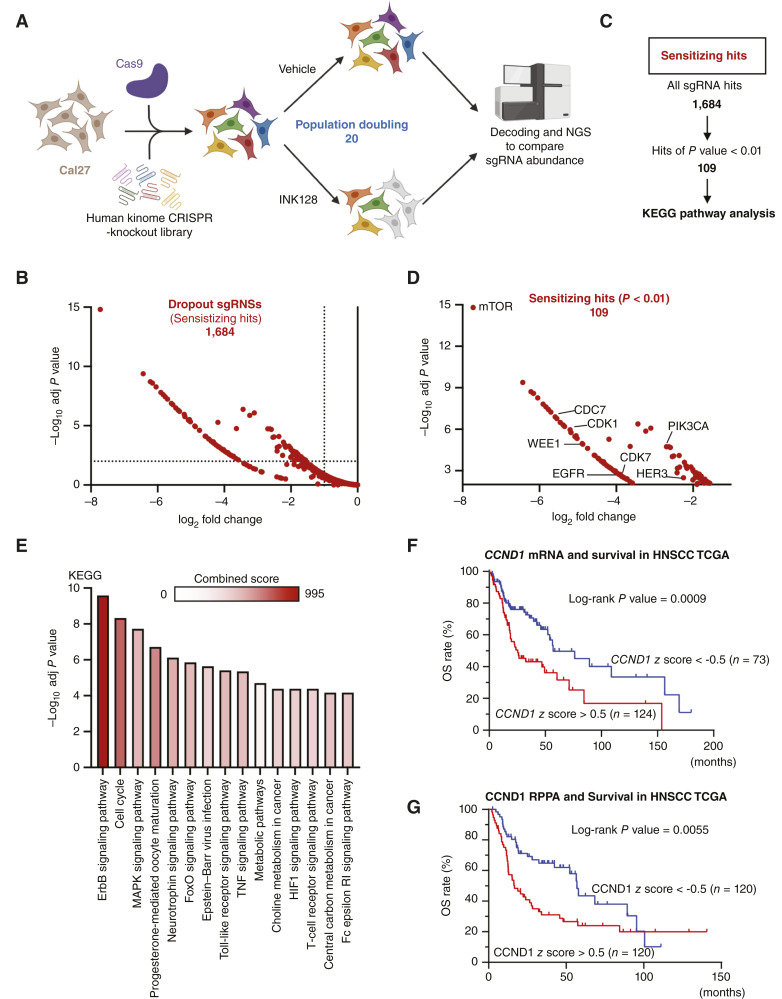
CRISPR screening identified the cell-cycle pathway as the synthetic lethal pathway for mTORis in HNSCC. **A,** Scheme for CRISPR screening. Cal27-Cas9 cells were infected with the human kinome CRIPSR-knockout library, targeting 763 genes consisting of four sgRNAs for each gene, and subjected to vehicle treatment or INK128 treatment. At population doubling 20, genomic DNA was extracted from cells, and PCR and NGS were performed. **B,** Volcano plot of all dropout sgRNA hits. **C,** Extraction sequence of sensitizing sgRNA hits. The sensitizing hits were extracted with *P* value < 0.01 and analyzed with KEGG pathway analysis. **D,** Selected 109 hits plot. The hits included genes related to PI3K/mTOR and cell-cycle pathways. **E,** KEGG pathway analysis for sensitizing hits. KEGG pathway analysis was applied for significant 109 hits. The top 15 pathways are represented, and the color intensity of the bar represents the combined score. **F,***CCND1* mRNA expression and OS in TCGA-HNSCC patients. Patients with high (*z*-score > 0.5, *n* = 124) and low (*Z*-score < 0.5, *n* = 73) expression of each mRNA were compared using the log-rank test. **G,** CCND1 RPPA expression and OS in TCGA-HNSCC patients. Patients with high (*z*-score > 0.5, *n* = 120) and low (*z*-score < 0.5, *n* = 120) expression of each mRNA were compared using the log-rank test. (Created with BioRender.com.)

To explore synthetic lethal targets and resistance mechanisms for mTORis in HNSCC, we took advantage of CRISPR screening. First, we generated Cas9-expressing Cal27 HNSCC cells (Cal27-Cas9; Supplementary Fig. S1A) and confirmed cutting efficiency using two different sgRNAs (gT1/gT2) targeting the AAVS locus. NGS for these cells showed 83.0% and 98.3% of nonhomologous end joining frequency for gT1 and gT2, respectively (Supplementary Fig. S1B), indicating that the cutting efficiency for Cal27–Cas9 was suitable to conduct the planned screening. As our purpose was to identify druggable targets and the kinome is the target of a large proportion of oncology-related drugs, we used a human kinome–wide CRISPR library, targeting 763 genes consisting of four sgRNAs for each gene (23). After infecting Cal27-Cas9 cells with the kinome-wide CRISPR library, we treated Cal27-Cas9-kinome cells with vehicle or mTORi until total population doubling reached 20 (Fig. 1A; Supplementary Fig. S1C). In this study, we applied INK128 (also known as MLN0128 and TAK-228), which is an mTOR ATP-competitive small-molecule inhibitor and reported to have excellent physiochemical properties (24, 25). After extracting DNA from these cells, we performed PCR to amplify the barcodes and NGS to identify depleted sgRNAs in mTORi-treated cells compared with vehicle-treated cells (Fig. 1A). Quality control analysis of the sgRNA screen is shown in Supplementary Fig. S1D and S1E, and the configuration of PinAPL-Py software used is included in Supplementary Table S1.

We show all dropout-sgRNAs in Fig. 1B and Supplementary Table S2. As a specificity control, none of the 98 nontargeting sgRNAs revealed any significant changes when comparing the control and INK128-treated cells (Supplementary Table S3). Next, hits with significant *P* values less than 0.01 were selected, and 109 sensitizing hits were identified (Fig. 1C; Supplementary Table S4). Remarkably, the most depleted gene was *mTOR*, which is consistent with the fact that we used a low-dose mTORi to identify synthetic lethal targets (Fig. 1D). We next applied pathway analysis for hit sgRNAs not only focusing on single sgRNAs but investigating hit sgRNAs as an integrated set of genes to find effective pathways to target. The 109–sensitizing hit set was analyzed with KEGG pathway analysis using Enrichr (https://maayanlab.cloud/Enrichr/; Fig. 1E; Supplementary Table S5; ref. 26). The results revealed significant enrichment of the ErbB signaling pathway and MAPK signaling pathway. Aligned with these findings, cotargeting ErbB signaling with an mTORi for HNSCC has been investigated (27, 28), including our combination study of cetuximab and rapamycin or everolimus (29), and our group reported by RNAi screening that MAPK signaling is synthetic lethal with the first-generation mTORi (rapamycin; ref. 30). Of interest, we also found a highly significant enrichment of cell-cycle pathways (Fig. 1E). As progression through the cell cycle is regulated by cyclins and CDKs, we first analyzed the association between cyclin expression and prognosis of HNSCC using The Cancer genome Atlas (TCGA) data. The high mRNA and protein expression of CCND1, which is encoded by the *CCND1* gene, is a worse prognostic factor for OS in patients with HNSCC (Fig. 1F and G), supporting an important role of cell-cycle signaling for HNSCC. These results prompted us to explore the possibility of cotargeting cell-cycle mechanisms and mTOR signaling in HNSCC.

### Combination of INK128 and palbociclib shows strong synergism in HNSCC cells *in vitro*

**Figure 2:**
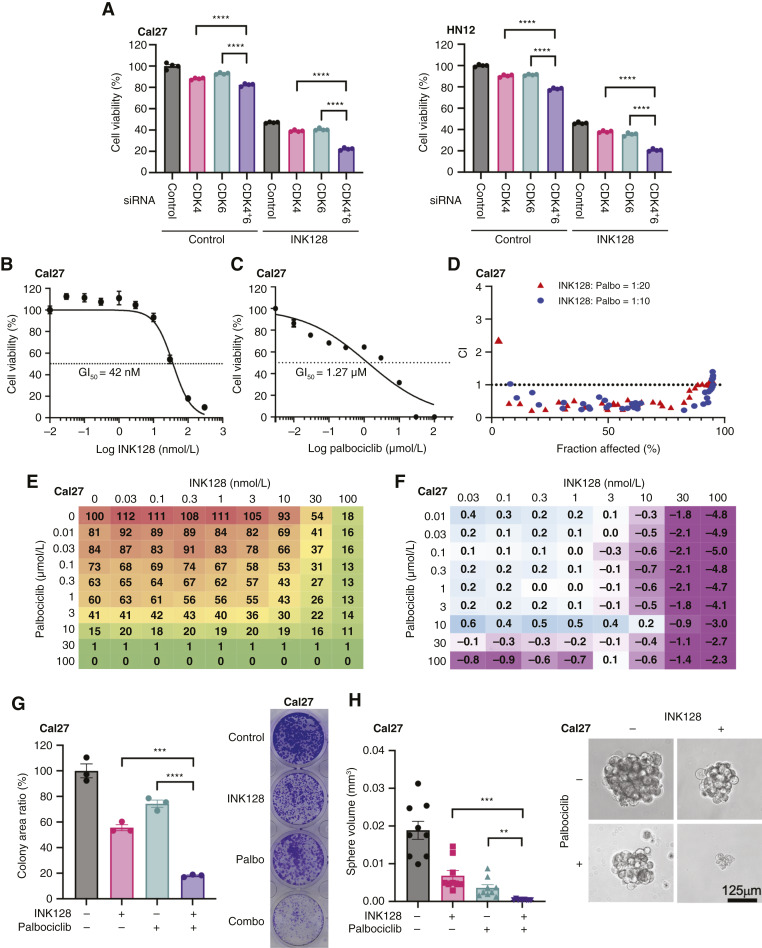
Combination of INK128 and palbociclib showed strong synergism in HNSCC cells *in vitro*. **A,** Cell viability of Cal27 and HN12 treated with siRNAs for 24 hours, followed by INK128 (30 nmol/L) treatment for another 48 hours, was measured (mean ± SEM, *n* = 4). **B,** Effect of INK128 on Cal27 HNSCC cells. INK128 potently blocked the cell viability with a GI_50_ of 42 nmol/L for Cal27 HNSCC cells (mean ± SEM, *n* = 3). **C,** Effect of palbociclib on Cal27 HNSCC cells. GI_50_ for palbociclib was 1.27 µmol/L for Cal27 cells (mean ± SEM, *n* = 3). **D,** Analysis for synergism between INK128 and palbociclib using the Chou–Talalay method for Cal27 cells. CI was below 1 for most percentage of fractions when cells were treated with INK128 and palbociclib at 1:10 or 1:20 concentration, respectively. **E,** Factorial dose matrix for INK128 and palbociclib. **F,** Analysis for synergism between INK128 and palbociclib using the Bliss model. Strong synergism was observed with relatively higher INK128 concentration than GI_50_. **G,** Colony formation abilities of Cal27 treated with INK128 (20 nmol/L) and/or palbociclib (0.4 µmol/L) were measured. Representative stained colony image of each treatment group. The colony area of each treatment group was compared relative to controls (mean ± SEM, *n* = 3). **H,** Orosphere formation abilities of Cal27 treated with INK128 (20 nmol/L) and/or palbociclib (0.4 µmol/L) were measured. Representative sphere image of each treatment group. Orosphere volume of each treatment group was compared (mean ± SEM, *n* = 9). ****, *P* < 0.0001; ***, *P* < 0.001; **, *P* < 0.01; *, *P* < 0.05; ns, nonsignificant. *P* value was determined using one-way ANOVA with the Tukey *post hoc* test in **G** and **H**. *P* value was determined using two-way ANOVA with the Tukey *post hoc* test in **A**. Palbo, palbociclib. (Created with BioRender.com.)

Although targeting CCND1 gene and protein levels may represent a therapeutic option in HNSCC, this may not be currently feasible, and instead, we focused on targeting their associated kinases, CDKs, as CDK inhibitors are already approved for other indications, thereby enhancing the translational potential of our studies. We first investigated the inhibitory effects of siRNA knockdown of CDK4 and CDK6, which are strongly associated with CCND (Supplementary Fig. S2A). Considering the enhanced clinical response of patients with HPV^−^ HNSCC (8), for these studies, we used representative HPV^−^ HNSCC cells (Cal27 and HN12). These cells harbor typical *TP53* mutations and exhibit persistent mTOR activation in the absence of *PIK3CA* mutations and *PTEN* genomic alterations that are more frequent in HPV-positive HNSCC lesions, thus reflecting the human HPV^−^ HNSCC oncogenome (10). Knockdown of CDK4 and/or CDK6 reduced cell viability only partially in HNSCC cells (Fig. 2A), which was increased by INK128 treatment. In this regard, individual CDK4 and CDK6 knockdown had limited impact on the response to INK128, but their growth-suppressive activity was significantly increased when the knockdown of both kinases was combined (Fig. 2A). This likely redundancy may explain why these kinases were not initially identified individually as targets in our CRISPR screen. In turn, this observation provided an opportunity to investigate the use of palbociclib, an FDA-approved cell cycle–targeted agent that can specifically inhibit CDK4/CDK6, to block cell-cycle signaling in combination with mTORi, and specifically in HPV^−^ HNSCC cells, as HPV-positive cells are refractory to CDK inhibition due to the viral oncoprotein E7 causing Rb degradation (31).

INK128 potently blocked the cell viability with a growth inhibition of 50% (GI_50_) of 42 nmol/L for Cal27 cells (Fig. 2B), and GI_50_ for palbociclib was 1.27 µmol/L for Cal27 cells (Fig. 2C). Similarly, GI_50_ for INK128 in HN12 was 28 nmol/L, and that for palbociclib was 0.85 µmol/L (Supplementary Fig. S2B and S2C). Next, we investigated the synergism between these drugs using the Chou–Talalay method (19). The fraction-affected CI plot showed a CI of below 1 for most percentage of fractions when cells were treated with 1:10 or 1:20 concentrations of INK128 and palbociclib, respectively (Fig. 2D). These data suggest a strong synergistic effect of this combination. In addition, we performed a factorial dose matrix combinatorial drug treatment with INK128 and palbociclib, supporting synergism for this combination (Fig. 2E). Furthermore, we analyzed synergism using the Bliss model (20), which suggested strong synergism with relatively higher INK128 concentration than GI_50_ (Fig. 2F). To confirm the synergism in another cell line, we used HN12 HNSCC cells. The CI was below 1 when used INK128 and palbociclib at 1:10 or 1:20 concentration, respectively, similar to Cal27 cells (Supplementary Fig. S2D). To further confirm the efficacy of combination therapy, we performed a colony formation assay and found that combination therapy significantly inhibited colony growth compared with treatment with INK128 or palbociclib alone (Fig. 2G; Supplementary Fig. S2E). To analyze the combination effect under conditions which are more reflective of cell growth in 3D, *in vivo* conditions, we tested orosphere assays, which allows for the propagation of cancer cells that retained stemness and self-renewal (32). INK128 and palbociclib significantly reduced the size of sphere formation in Cal27 and HN12 cells, and coadministration of these two drugs could significantly block the sphere formation (Fig. 2H; Supplementary Fig. S2F). These data suggest the possibility of using the combination of INK128 and palbociclib for the treatment of HNSCC.

### Upregulation of CCNE1 by palbociclib confers resistance to palbociclib, which can be reverted by INK128

**Figure 3:**
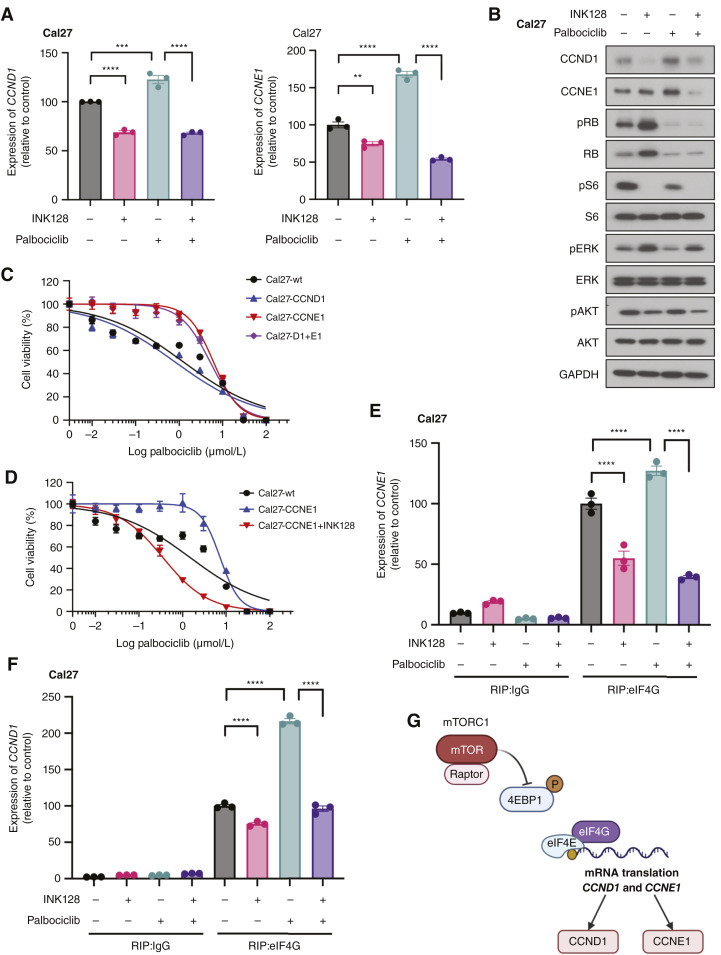
Upregulation of CCNE1 by palbociclib confers resistance to palbociclib, which can be reverted by INK128. **A,** Relative mRNA levels of *CCND1* and *CCNE1* in Cal27 treated with INK128 and/or palbociclib for 24 hours. **B,** Signaling change with INK128 and/or palbociclib treatment. Cal27 cells were treated with 50 nmol/L of INK128, 1 µmol/L of palbociclib, or both for 48 hours after serum starvation overnight and were analyzed for indicated proteins by Western blotting. **C,** Cell viability of Cal27 cells treated with palbociclib. Comparison of wt, overexpressing CCND1, CCNE1, and both (mean ± SEM, *n* = 3). **D,** Cell viability of Cal27 cells treated with palbociclib. Comparison of wt, overexpressing CCNE1, and CCNE1+INK128 treatment (mean ± SEM, *n* = 3). **E,** eIF4G binding assay with INK128 and/or palbociclib treatment for *CCNE1*. Proteins from each treated Cal27 were immunoprecipitated by eIF4G. RNA was extracted from the IP product, and expression of CCNE1 was determined by qPCR (mean ± SEM, *n* = 3). **F,** eIF4G binding assay with INK128 and/or palbociclib treatment for *CCND1*. Proteins from each treated Cal27 were immunoprecipitated by eIF4G. RNA was extracted from the IP product, and expression of CCND1 was determined by qPCR (mean ± SEM, *n* = 3). **G,** Schema for CCND1 and CCNE1 mRNA translation. mTORC1 phosphorylates 4EBP1 and promotes mRNA translation. ****, *P* < 0.0001; ***, *P* < 0.001; **, *P* < 0.01; *, *P* < 0.05; ns, nonsignificant. *P* value was determined using one-way ANOVA with the Tukey *post hoc* test in **A**. *P* value was determined using two-way ANOVA with the Tukey *post hoc* test in **E** and **F**. (Created with BioRender.com.)

To investigate the mechanism for the synergism between INK128 and palbociclib in HNSCC, we explored changes in signaling components and cell-cycle mechanisms. Based on our results above, we first focused on CCND1 and CCNE1 associated with CDK4/6. The qPCR result showed that *CCND1* and *CCNE1* mRNA expressions were significantly increased by palbociclib treatment in Cal27 and HN12 (Fig. 3A; Supplementary Fig. S3A). On the other hand, *CCND1* and *CCNE1* expressions were significantly decreased with INK128 alone and combination treatment. Next, we performed Western blotting to exam the protein expression. As expected, INK128 could effectively inhibit PI3K/mTOR activation as judged by pAKT and pS6 expression levels in Cal27 and HN12 (Fig. 3B; Supplementary Fig. S3B). As for the cell cycle, INK128 increased phosphorylation of retinoblastoma protein (RB) and decreased CCND1 expression. Although INK128 suppresses the AKT/mTOR pathway, it promotes RB phosphorylation and activates cell-cycle pathways. In contrast, palbociclib treatment prevented phosphorylation of RB and caused upregulation of CCND1 and CCNE1. We hypothesized that CCND1 and CCNE1 activation could represent a mechanism for palbociclib resistance in HNSCC, considering recent clinical data showing high *CCNE1* as worse clinical outcome for palbociclib-treated patients in breast cancer (33, 34). In this regard, we engineered HNSCC cell lines which stably overexpress CCND1 and CCNE1 individually and together (Supplementary Fig. S3C). This approach revealed increased resistance to palbociclib in Cal27-CCNE1 cells compared with Cal27–wild-type (wt) cells (Fig. 3C). However, no significant resistance to palbociclib was observed in Cal27-CCND1 cells. CCND1/E1-overexpressing Cal27 cells were also resistant; however, the resistance was similar to that of CCNE1–Cal27 cells, which suggests that no additional resistance was conferred by CCND1 overexpression. Similar results were confirmed using HN12-wt, HN12-CCND1, HN12-CCNE1, and HN12-CCND1/E1 and palbociclib (Supplementary Fig. S3D). These data indicate that CCNE1 overexpression may represent one of the mechanisms of resistance to palbociclib in HNSCC.

Remarkably, although we observed upregulation of CCNE1 after treatment with palbociclib in HNSCC cells, the addition of INK128 together with palbociclib could revert this overexpression and downregulated CCNE1 (Fig. 3B; Supplementary Fig. S3B). Furthermore, the resistance of Cal27-CCNE1 and HN12-CCNE1 to palbociclib in terms of cell viability was completely abolished by the addition of INK128 (Fig. 3D; Supplementary Fig. S3E). These results indicate that INK128 treatment suppresses palbociclib-induced CCNE1 elevation and prevents resistance acquisition. To gain a mechanistic insight into this process, we built on our previous observations that blockade of mTOR by INK128 leads to dephosphorylation of 4EBP1, which in turn reduces eIF4E and eIF4G binding, resulting in reduced mRNA translation of proliferating proteins (24). This was confirmed in the present study (Supplementary Fig. S3F). Next, we performed RIP assays to directly investigate the binding of endogenous eIF4G to *CCND1* and *CCNE1* mRNAs. Indeed, INK128 treatment reduced the binding of eIF4G to *CCNE1* mRNA in Cal27 and HN12 cells (Fig. 3E; Supplementary Fig. S3G). Binding of eIF4G to *CCND1* mRNA was reduced by INK128 treatment in Cal27 but not in HN12 (Fig. 3F; Supplementary Fig. S3H), which suggests cancer heterogeneity in this response and a more general impact on *CCNE1*. On the other hand, palbociclib significantly increased binding of eIF4G to the mRNA for *CCND1* and *CCNE1* in both Cal27 and HN12 cells. Thus, we hypothesized that INK128 could revert the overexpression of CCNE1 caused by palbociclib treatment by reducing binding of eIF4G and *CCNE1*. As shown in Fig. 3E; Supplementary Fig. S3G, combination treatment with INK128 and palbociclib potently reduced *CCNE1* mRNA binding to eIF4G compared with control or palbociclib treatment. These data suggest that CCNE1 activation or overexpression represents one of the resistance mechanisms to palbociclib and that mTOR acts upstream of CCNE1, controlling its mRNA translation (Fig. 3G). Together, these data provide a rationale for the combination therapy of INK128 and palbociclib for HNSCC.

### Combination therapy with INK128 and palbociclib is effective against HNSCC xenograft models

**Figure 4:**
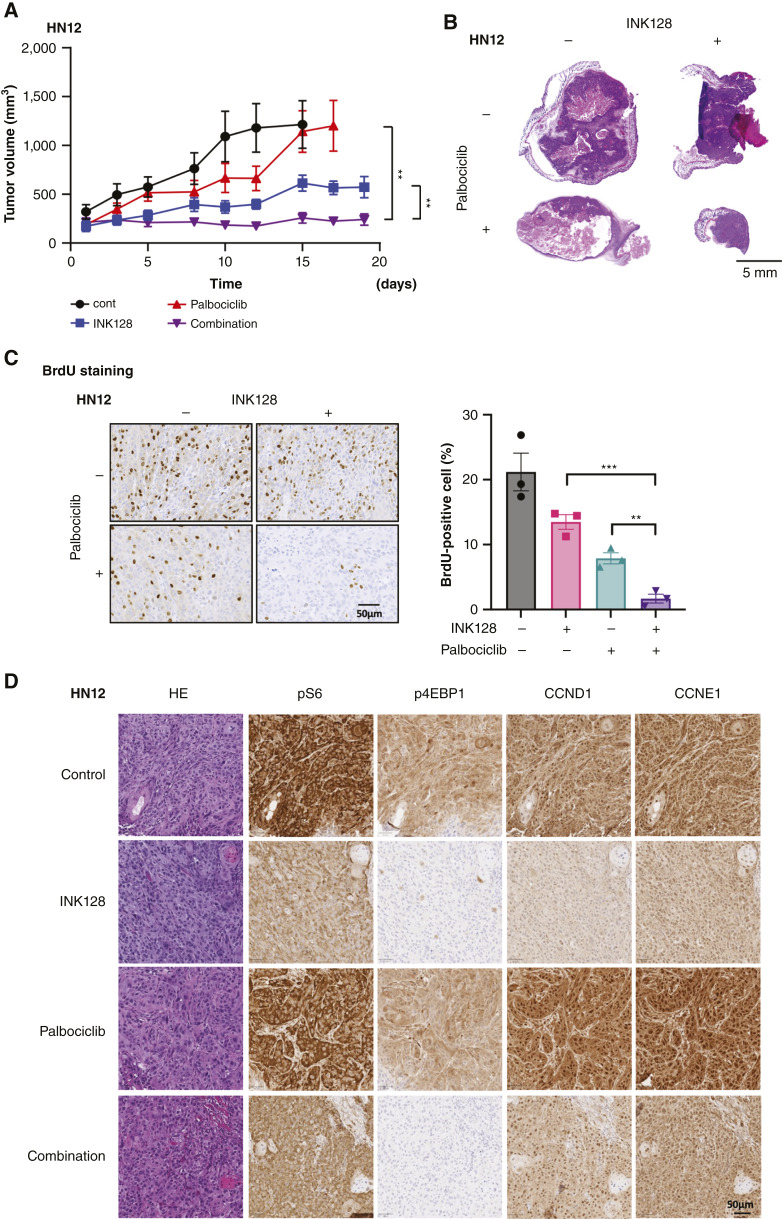
Combination therapy with INK128 and palbociclib is effective against HNSCC xenografts. **A,** Tumor growth curve for HN12 xenografts treated with INK128, palbociclib, and combination (mean ± SEM, *n* = 10). **B,** Hematoxylin and eosin staining of HN12 xenograft tumors. **C,** BrdU staining for HN12 xenograft tumors. The percentage of BrdU-positive cells was compared by treatment group (mean ± SEM, *n* = 3). **D,** Representative IHC staining images of HN12 xenografts (strongly magnified). Tumors were stained with hematoxylin–eosin (HE), pS6, p4EBP1, CCND1, and CCNE1. ****, *P* < 0.0001; ***, *P* < 0.001; **, *P* < 0.01; *, *P* < 0.05, ns, nonsignificant. *P* value was determined using one-way ANOVA with the Tukey *post hoc* test in **A** and **C**. (Created with BioRender.com.)

Next, we asked if this combination of INK128 and palbociclib is effective *in vivo*. Using Cal27 and HN12 xenograft models, we started treatment with INK128, palbociclib, or combination after tumors were established. Because high frequency of myelosuppression has been reported for palbociclib in clinical trials (35), we used relatively low-dose palbociclib for this *in vivo* study. In our Cal27 xenograft model, INK128 or palbociclib treatment as a single agent did not inhibit tumor growth, but the combination of these drugs significantly inhibited tumor growth (Supplementary Fig. S4A). As for the HN12 xenograft, palbociclib did not inhibit tumor growth, and INK128 was relatively effective as a single agent, but combination therapy had a significantly stronger effect than single agents (Fig. 4A). The hematoxylin and eosin staining of these tumors showed that mTOR inhibition together with palbociclib caused tumor collapse with smallest residual tumor masses at the end of the treatment (Fig. 4B; Supplementary Fig. S4B). To assess the inhibition of proliferation *in vivo*, we used BrdU staining for tumors with short-term treatment of palbociclib, INK128, or combination. The combination therapy demonstrated the lowest percentage of BrdU-positive cells in both Cal27 and HN12 xenografts, which indicates strong inhibition of cell proliferation in coadministered tumors (Fig. 4C; Supplementary Fig. S4C). In addition, we used IHC to confirm protein expression in the tumors (Fig. 4D; Supplementary Fig. S4D). INK128 treatment decreased expression of phospho-S6 and phospho-4EBP1, whereas palbociclib treatment increased the expression of CCND1 and CCNE1. In contrast, these increases were suppressed by INK128 in the combination treatment. Furthermore, despite only 5 days of treatment, combination therapy induces tumor collapse (Supplementary Fig. S4D). These results indicate that even in *in vivo*, the cyclin upregulation induced by palbociclib is rescued by mTORi, thereby displaying a strong synergistic effect.

## Discussion

The frequent genomic alterations in *CDKN2A* and *CCND1* in HPV^−^ clinical HNSCC cases suggest that there is a strong rationale to target CDK4/6 to inhibit tumor progression in HNSCC. Several selective CDK4/6 inhibitors are available in the clinic, such as abemaciclib, ribociclib, and palbociclib. Among them, palbociclib is the first FDA-approved CDK4/6-specific inhibitor, inducing G1 arrest, with a concomitant reduction of phosphorylation of the Rb protein (36). It is approved for advanced or metastatic, hormone receptor–positive, and HER2-negative breast cancer, in combination with endocrine therapy (34, 37). For HPV^−^negative HNSCC, several clinical trials have been conducted using CDK4/6 inhibitors. In selected patients with *CDKN2A*-altered HNSCC, palbociclib monotherapy showed modest antitumor activity (4). In addition, in the PALATINUS study, the combination of palbociclib and cetuximab did not prolong the OS in unselected patients (5). To strengthen the antitumor activity of palbociclib in HNSCC, novel strategies are needed. In the subgroup analysis in PALATINUS patients, trends for better OS were observed in patients with *CDKN2A* mutations or CDK4/6 amplification but in the absence of *PIK3CA* alterations (5). Consistent with these data, basic studies showed that *PIK3CA*-mutant HNSCC cells are less responsive to palbociclib (38). These results are consistent with the results of our study showing that mTORi and palbociclib could have beneficial combinatory effects on HNSCC.

The therapeutic potential of mTORis for HNSCC has been extensively studied. Our group pioneered the use of rapamycin as a single agent to treat HNSCC xenografts (6). In this early study, we showed that phosphorylated S6, the most downstream target of the Akt–mTOR pathway, is frequently accumulated in HNSCC clinical specimens. Furthermore, we used rapamycin to treat four different types of HNSCC xenografts, resulting in tumor regression. Following this study, several groups have reported the effectiveness of mTORis for HNSCC (39–41). In turn, these analyses from basic research led to multiple clinical trials including single-agent mTORis or combined treatment with mTORi and other agents (9, 42–45). Our group has recently shown the efficacy of rapamycin as monotherapy for previously untreated patients. A 21-day treatment for 16 patients with rapamycin resulted in one complete response, three partial responses, and 12 stable diseases, supporting the potential role of mTORis for HNSCC (7). Furthermore, clinical trials with administration of metformin, which has been shown to regulate mTOR via AMPK, to premalignant lesions of HNSCC have been conducted, and they show promising results as judged by pathologic responses (46). Similarly, we have recently shown that mTOR inhibition with everolimus in the adjuvant setting after definitive treatment of locally advanced HNSCC lesions reduces significantly tumor relapse, specifically in HPV^−^ cases (8). However, a clinical trial targeting mTOR in heavily pretreated patients with HNSCC did not show clinical benefit with everolimus (9). These findings suggest that previous treatments may cause genetic alterations and epigenetic changes in cancer cells; consequently, more complicated mechanisms driving cell growth may be active in these lesions when compared with the use of mTORis in newly diagnosed HNSCC cases or as an adjuvant postsurgery and/or radiation. In addition, in these early clinical trials, mainly three mTORis were used: rapamycin (sirolimus), everolimus, and temsirolimus. These three mTORis are often referred to as first-generation mTORis, blocking only mTORC1. In our study, we used INK128, which is a second-generation mTORi that binds to the ATP-binding site of mTOR and inhibits the catalytic activity of both mTORC1 and mTORC2 without inhibiting other kinases (25). In this regard, INK128 is different from previous mTORis, and the antitumor effect of second-generation mTORis is promising (47).

**Figure 5:**
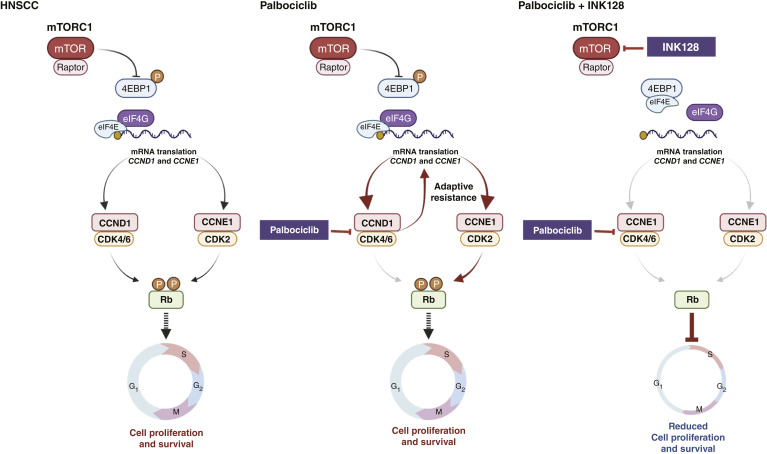
Schematic representation of the mechanism for combination treatment with INK128 and palbociclib. See “Discussion” for details. (Created with BioRender.com.)

To overcome potential mechanisms limiting the response to mTORis, we hypothesized that the administration of mTORis to HNSCC combined with targeting agents suppressing resistance pathways may provide better outcomes. In this study, we applied an unbiased approach to find synthetic lethal and resistance targets for INK128 and showed that the cell-cycle pathway can be a synthetic lethal target with INK128. Xenograft experiments using human HNSCC cells showed promising results with the coadministration of INK128 and palbociclib. Mechanistically, we showed that INK128 could inhibit the adaptive accumulation of CCNE1 caused by palbociclib. Because INK128 blocks mTORC1 and mTORC2, it can inhibit phosphorylation of 4EBP1 strongly, which in turn reactivates the tumor-suppressive activity of 4EBP1 (24, 25). Dephosphorylated 4EBP1 associates with eIF4E and inhibits binding between eIF4E and eIF4G, resulting in reduced translation of mRNAs that are essential to cell proliferation for tumor (24). In this case, one of the eIF4G-binding mRNAs reduced by INK128 is *CCNE1*. This may explain the reduced level of CCNE1 protein after INK128 and the efficacy of combination therapy with INK128 and palbociclib (Fig. 5).

Our findings may now provide a mechanistic framework on the interplay between CDK4/CDK6 blockade and mTORi resulting in increased tumor control and prevent the acquisition of palbociclib resistance. Aligned with this perspective, recent studies suggest that enhanced *CCNE1* mRNA expression levels in patients with breast cancer are associated with resistance to palbociclib (33, 48), which was recapitulated by our current findings supporting that CCNE1 overexpression is sufficient to induce palbociclib resistance in HNSCC cells. Similarly, the PI3K/mTOR pathway was found to be overactivated in palbociclib-resistant breast cancer cells, with increased levels of CCND1 and CDK4 translation that could be reverted by PI3K/mTOR inhibition (49), and palbociclib-based high-throughput combination drug screens showed a significant synergistic effect when palbociclib was combined with PI3K, EGFR, or MEK inhibitors in HNSCC (50). Furthermore, in *PIK3CA*-mutant HNSCC cells, a combination of a PI3K/mTORi and palbociclib was reported to be effective in xenograft tumors (38), albeit by a poorly understood mechanism. These results support that the use of palbociclib alone has limited activity in HNSCC, likely because of the rapid acquisition of adaptive resistance through a positive feedback loop resulting in increased CCNE1 expression, which can be prevented by the concomitant mTOR pathway blockade (Fig. 5).

In summary, our unbiased genetic library screen approach revealed that concomitant mTOR blockade reverts the adaptive resistance to palbociclib. Specifically, CCNE1 overexpression caused by palbociclib can be abolished by coadministration of INK128. Ultimately, our findings may provide a novel strategy for patients with HPV^−^ HNSCC by cotargeting mTOR and key cell cycle–regulating molecules, which can also have an impact in multiple cancer types that fail to respond to CDK4/6 inhibitors as single agents.

## Supplementary Material

Supplementary Table 1 Configuration of PinAPL-Py software

Supplementary Tables 2 -5 List of dropout sgRNAs, non-targeting control sgRNAs, selected sensitizing genes, and KEGG pathway analysis of sensitizing genes

Supplementary Figure 1 CRISPR screening identified cell cycle pathway as synthetic lethal pathway for mTORi in HNSCC

Supplementary Figure 2 Combination of INK128 and palbociclib showed strong synergism in HNSCC cells in vitro

Supplementary Figure 3 Upregulation of CCNE1 by palbociclib confers resistance to palbociclib, which can be reverted by INK128

Supplementary Figure 4 Combination therapy with INK128 and palbociclib is effective against HNSCC xenograft
