# Supplementary material for: A Kinome-Wide Synthetic Lethal CRISPR/Cas9 Screen Reveals That mTOR Inhibition Prevents Adaptive Resistance to CDK4/CDK6 Blockade in HNSCC
Source: Cancer Res Commun. 2024 Jul 29;4(7):1850–62. doi: 10.1158/2767-9764.CRC-24-0247 (PMC11284272; doi:10.1158/2767-9764.CRC-24-0247)
Supplement: Supplementary Table 1 — Configuration of PinAPL-Py software [file crc-24-0247_supplementary_table_1_suppst1.pdf]

Supplementary Table S1. Configuration of PinAPL-Py software

Required
ScreenType: 'depletion'
LibFilename: 'Brunello_kinome_guides1-4.tsv'

Library Parameters
LibFilename: 'Brunello_kinome_guides1-4.tsv'
seq_5_end: 'TCTTGTGGAAAGGACGAAACACCN'
NonTargetPrefix: 'Non-Targeting'
NumGuidesPerGene: 4

Alignment
sgRNALength: 20
CutErrorTol: 0.1
AS_min: 40
Theta: 2
L_bw: 11
N_bw: 1
i_bw: 'S,1,0.75'
AlnOutput: 'Delete'
keepCutReads: False
delta: 1
R_min: 20

Read Counting
Normalization: 'cpm'
Cutoff: 0
RoundCount: False
repl_avg: 'median'

Gene Ranking
GeneMetric: 'aRRA'
Np: 1000
P_0: 0.01
thr_STARS: 10

```
# Statistical Significance
alpha_s: 0.01
alpha_g: 0.01
padj: 'fdr_bh'
p_overdisp: 0.01

# Sample Clustering
ClusterBy: 'variance'
TopN: 25

# Visualization
dotsize: 10
TransparencyLevel: 0.1
scatter_annotate: False
ShowNonTargets: False
logbase: 10
width_p: 800
height_p: 800
fontsize_p: 14
marginsize: 10
max_q: 95
svg: True
dpi: 300
HitListFormat: 'tsv'

WorkingDir: '/workingdir/'
DataDir: '/workingdir/Data/'
TempDataDir: '/workingdir/TempData/'
LibDir: '/workingdir/Library/'
IndexDir: '/workingdir/Library/Bowtie2_Index/'
ScriptsDir: '/opt/PinAPL-Py/Scripts/'
AlignDir: '/workingdir/Alignments/'
AnalysisDir: '/workingdir/Analysis/'
TrimLogDir: '/workingdir/Analysis/Read_Trimming'
HitDir: '/workingdir/Analysis/sgRNA_Rankings'
```

GeneDir: '/workingdir/Analysis/Gene_Rankings'
ControlDir: '/workingdir/Analysis/Control/'
HeatDir: '/workingdir/Analysis/Heatmap/'
AlnQCDir: '/workingdir/Analysis/Alignment_Statistics/'
CountQCDir: '/workingdir/Analysis/ReadCount_Statistics/'
ScatterDir: '/workingdir/Analysis/ReadCount_Scatterplots/'
HiLiteDir: '/workingdir/Analysis/ReadCount_Scatterplots/Highlighted_Genes/'
CorrelDir: '/workingdir/Analysis/Replicate_Correlation/'
HiLiteDir2: '/workingdir/Analysis/Replicate_Correlation/Highlighted_Genes'
EffDir: '/workingdir/Analysis/sgRNA_Efficacy/'
DepthDir: '/workingdir/Analysis/Read_Depth/'
SeqQCDir: '/workingdir/Analysis/Sequence_Quality/'
pvalDir: '/workingdir/Analysis/p-values/'
LogFileDir: '/workingdir/Analysis/Log_File/'
bw2Dir: '/usr/bin/'
CutAdaptDir: '/root/.local/bin/'
STARSDir: '/opt/PinAPL-Py/Scripts/STARS_mod/'

SanityScript: 'CheckCharacters'
IndexScript: 'BuildLibraryIndex'
LoaderScript: 'LoadDataSheet'
ReadDepthScript: 'PlotNumReads'
SeqQCScript: 'CheckSequenceQuality'
TrimScript: 'TrimReads'
AlignScript: 'AlignReads'
NormalizeScript: 'NormalizeReadCounts'
AverageCountsScript: 'AverageCounts'
StatsScript: 'AnalyzeReadCounts'
ControlScript: 'AnalyzeControl'
sgRNARankScript: 'FindHits'
GeneRankScript: 'RankGenes'
CombineScript: 'CombineGeneRanks'
ScatterScript: 'PlotCounts'
ReplicateScript: 'PlotReplicates'
ClusterScript: 'PlotHeatmap'
