# Supplementary material for: A Kinome-Wide Synthetic Lethal CRISPR/Cas9 Screen Reveals That mTOR Inhibition Prevents Adaptive Resistance to CDK4/CDK6 Blockade in HNSCC
Source: Cancer Res Commun. 2024 Jul 29;4(7):1850–62. doi: 10.1158/2767-9764.CRC-24-0247 (PMC11284272; doi:10.1158/2767-9764.CRC-24-0247)
Supplement: Supplementary Figure 1 — CRISPR screening identified cell cycle pathway as synthetic lethal pathway for mTORi in HNSCC [file crc-24-0247_supplementary_figure_1_suppsf1.pdf]

Supplementary Figure S1

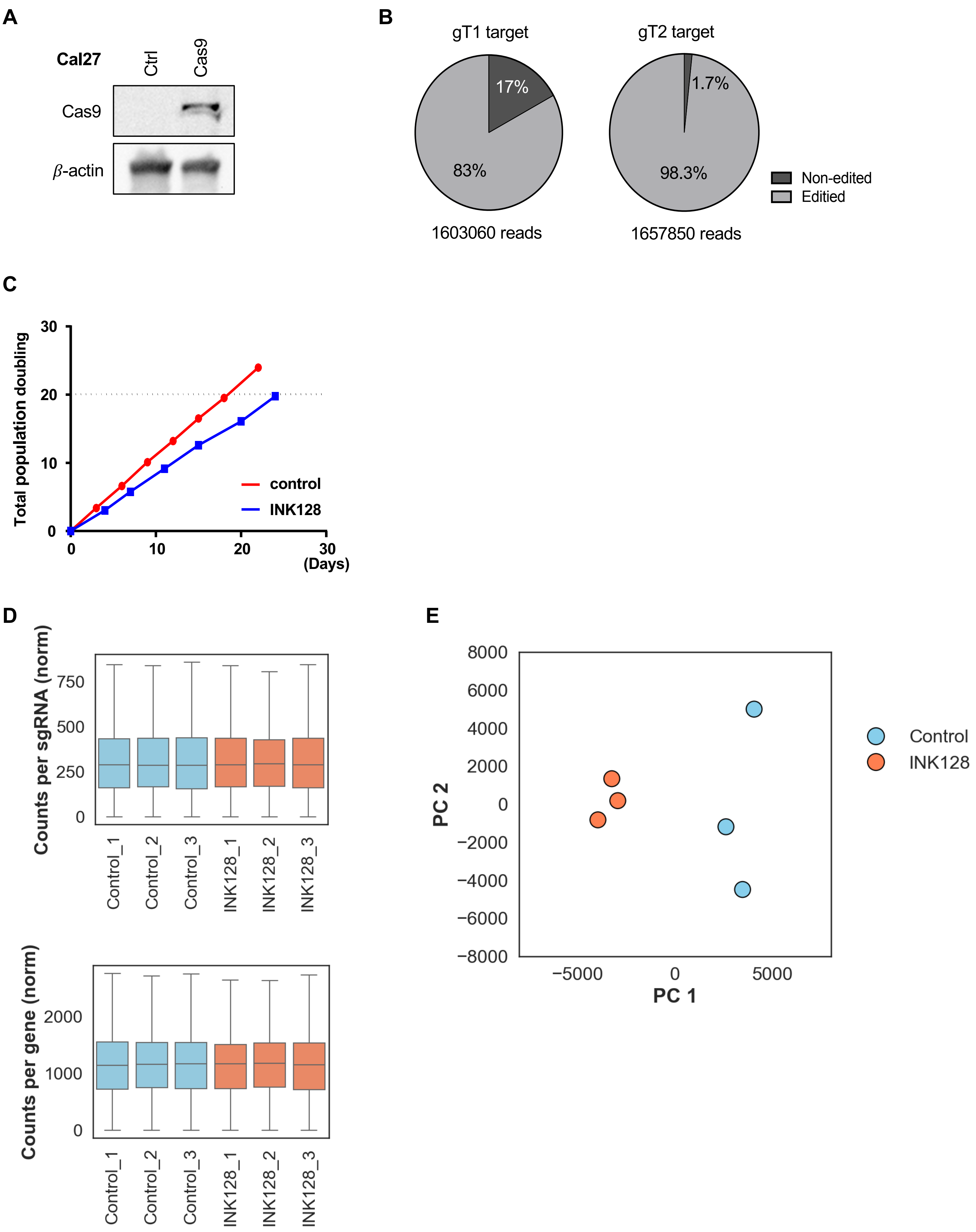

Supplementary Figure S1. CRISPR screening identified cell cycle pathway as synthetic lethal pathway for mTORi in HNSCC

A. Cas9 expression of Cal27 cells. Cas9 expression was confirmed by Western blots. **B.** Quantification of the AAVS1 locus editing frequency. NGS showed 83.0% and 98.3% of non-homologous end joining (NHEJ) frequency for gT1 and gT2, respectively. **C.** Total PD difference between two groups. At day 18, control cells proliferated to total PD of 20, and INK128 treated cells proliferated to total PD 20 at day 24. **D.** As a quality control, the analysis of normalized sgRNAs and gene counts in the control and INK128 treated groups with approximately 250 and 1000 reads per million (RPM), demonstrated good sequencing depth and consistent patterns within each group. **E.** The principal component analysis (PCA) plot illustrates the separation between control and INK128, with each point representing a sample. Control samples cluster tightly together, indicating similar profiles, while INK128 samples form a distinct cluster, suggesting a treatment-induced profile shift. The clear separation along PC1 indicates a significant effect of INK128 treatment, differentiating it from the control.
