# Supplementary material for: A Kinome-Wide Synthetic Lethal CRISPR/Cas9 Screen Reveals That mTOR Inhibition Prevents Adaptive Resistance to CDK4/CDK6 Blockade in HNSCC
Source: Cancer Res Commun. 2024 Jul 29;4(7):1850–62. doi: 10.1158/2767-9764.CRC-24-0247 (PMC11284272; doi:10.1158/2767-9764.CRC-24-0247)
Supplement: Supplementary Figure 2 — Combination of INK128 and palbociclib showed strong synergism in HNSCC cells in vitro [file crc-24-0247_supplementary_figure_2_suppsf2.pdf]

Supplementary Figure S2

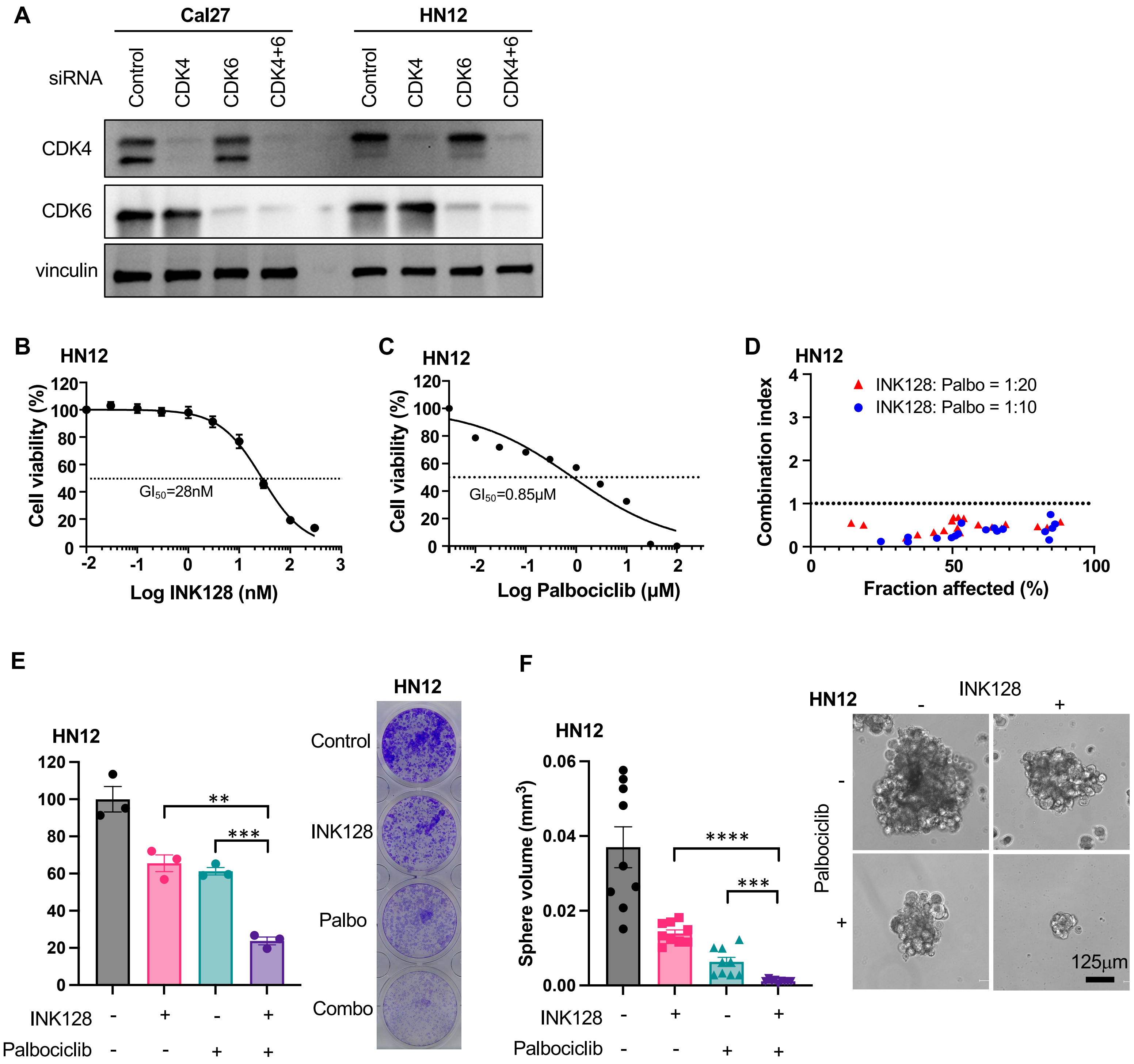

Supplementary Figure S2. Combination of INK128 and palbociclib showed strong synergism in HNSCC cells in vitro

A. Cal27 and HN12 treated with siRNAs for 48 hours were analyzed by western blotting to confirm the effect of siRNA. **B.** The effect of INK128 on HN12 HNSCC cells. GI_{50} for INK128 was 28 nM for HN12 HNSCC cells (mean ± SEM, n = 3). **C.** The effect of palbociclib on HN12 HNSCC cells. GI_{50} for palbociclib was 0.32 μM for HN12 cells (mean ± SEM, n = 3). **D.** Analysis for synergism between INK128 and palbociclib by Chou-Talalay method for HN12 cells. CI was below 1 for most percentage of fraction when cells were treated with 1:10 or 1:20 concentration of INK128 and palbociclib, respectively. **E.** Colony formation abilities of HN12 treated with INK128 (20nM) and/or palbociclib (0.4μM) were measured. Representative stained colony image of each treatment group. Colony area of each treatment group was compared relative to controls (mean ± SEM, n = 3). **F.** Orosphere formation abilities of HN12 treated with INK128 (20nM) and/or palbociclib (0.4μM) were measured. Representative sphere image of each treatment group. Orosphere volume of each treatment group was compared (mean ± SEM, n = 9). ****P < 0.0001, ***P < 0.001, **P < 0.01, *P < 0.05, ns = non-significant. p-value was determined by one-way ANOVA with Tukey's post hoc test in Supplementary Figure S2E and S2F.
