# Supplementary material for: A Kinome-Wide Synthetic Lethal CRISPR/Cas9 Screen Reveals That mTOR Inhibition Prevents Adaptive Resistance to CDK4/CDK6 Blockade in HNSCC
Source: Cancer Res Commun. 2024 Jul 29;4(7):1850–62. doi: 10.1158/2767-9764.CRC-24-0247 (PMC11284272; doi:10.1158/2767-9764.CRC-24-0247)
Supplement: Supplementary Figure 3 — Upregulation of CCNE1 by palbociclib confers resistance to palbociclib, which can be reverted by INK128 [file crc-24-0247_supplementary_figure_3_suppsf3.pdf]

Supplementary Figure S3

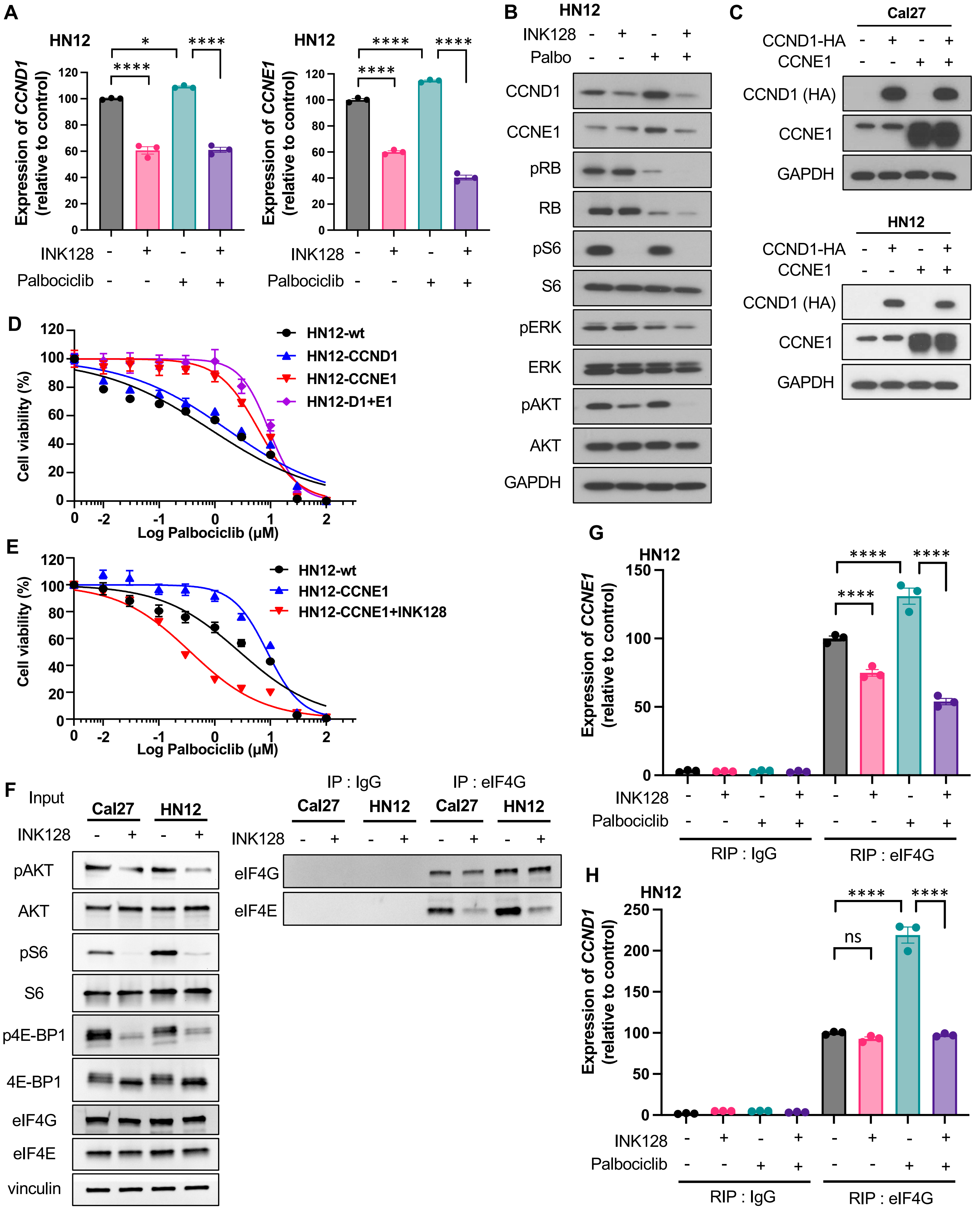

Supplementary Figure S3. Upregulation of CCNE1 by palbociclib confers resistance to palbociclib, which can be reverted by INK128

A. Relative mRNA levels of *CCND1* and *CCNE1* in HN12 treated with INK128 and/or palbociclib for 24 hours. **B.** Signaling change with INK128 and/or palbociclib treatment. HN12 was treated with 50 nM of INK128, 1μM of palbociclib, or both for 48 hours after serum starvation overnight, and were analyzed for indicated proteins by western blotting. **C.** Establishment of stable cell lines with CCND1 and CCNE1 overexpression. Cal27-CCND1, Cal27-CCNE1, Cal27-CCND1+CCNE1, HN12-CCND1, HN12-CCNE1, and HN12-CCND1+CCNE1 were confirmed by western blotting. **D.** Cell viability of HN12 cells treated with palbociclib. Comparison of wild type, overexpressing CCND1, CCNE1 and both (mean ± SEM, n = 3). **E.** Cell viability of HN12 cells treated with palbociclib. Comparison of wild type, overexpressing CCNE1, CCNE1+INK128 treatment (mean ± SEM, n = 3). **F.** Cal27 and HN12 cells were treated with INK128 (30nM) for 24 hours, and cell lysates were collected and immunoprecipitated with eIF4G. The input proteins and IP products were analyzed for indicated proteins by western blotting. **G.** eIF4G binding assay with INK128 and/or palbociclib treatment for *CCNE1*. Proteins from each treated HN12 were immunoprecipitated by eIF4G. RNA was extracted from the IP product and expression of CCNE1 was determined by qPCR (mean ± SEM, n = 3). **H.** eIF4G binding assay with INK128 and/or palbociclib treatment for *CCND1*. Proteins from each treated HN12 were immunoprecipitated by eIF4G. RNA was extracted from the IP product and expression of CCND1 was determined by qPCR (mean ± SEM, n = 3). ****$P < 0.0001$, ***$P < 0.001$, **$P < 0.01$, *$P < 0.05$, ns = non-significant. p-value was determined by one-way ANOVA with Tukey's post hoc test in Supplementary Figure S3A. p-value was determined by two-way ANOVA with Tukey's post hoc test in Supplementary Figure S3G and S3H.
