# Supplementary material for: A Kinome-Wide Synthetic Lethal CRISPR/Cas9 Screen Reveals That mTOR Inhibition Prevents Adaptive Resistance to CDK4/CDK6 Blockade in HNSCC
Source: Cancer Res Commun. 2024 Jul 29;4(7):1850–62. doi: 10.1158/2767-9764.CRC-24-0247 (PMC11284272; doi:10.1158/2767-9764.CRC-24-0247)
Supplement: Supplementary Figure 4 — Combination therapy with INK128 and palbociclib is effective against HNSCC xenograft [file crc-24-0247_supplementary_figure_4_suppsf4.pdf]

Supplementary Figure S4

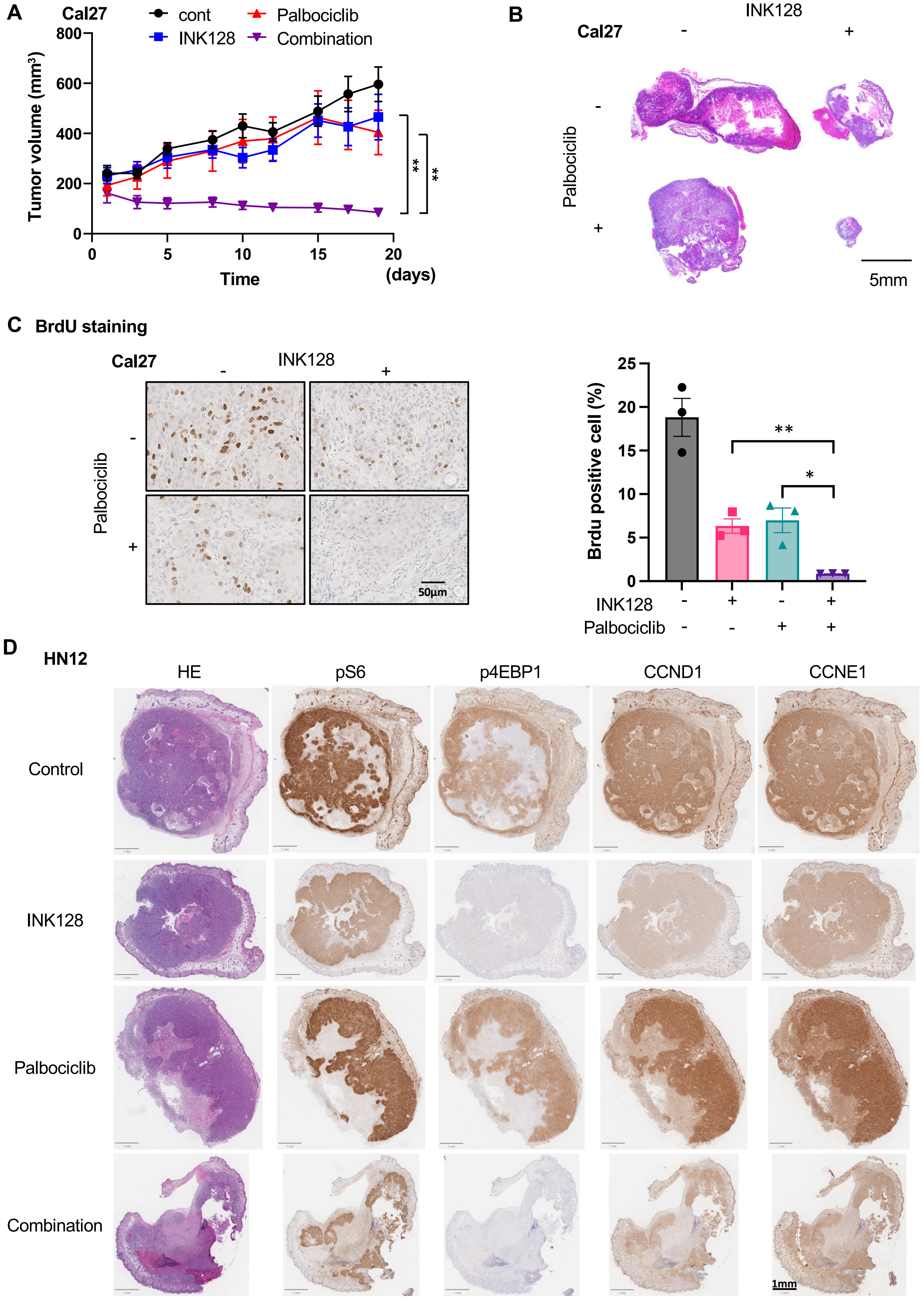

Supplementary Figure S4. Combination therapy with INK128 and palbociclib is effective against HNSCC xenograft

A. Tumor growth curve for HN12 xenograft with INK128, palbociclib, and combination (mean ± SEM, n = 10). **B.** H&E staining of HN12 xenograft tumors. **C.** BrdU staining for HN12 xenograft tumors. The percentage of BrdU-positive cells was compared by treatment group (mean ± SEM, n = 3). **D.** Representative IHC staining images of HN12 xenografts. Tumors were stained with HE, pS6, p4EBP1, CCND1 and CCNE1. ****$P < 0.0001$, ***$P < 0.001$, **$P < 0.01$, *$P < 0.05$, ns = non-significant. p-value was determined by one-way ANOVA with Tukey's post hoc test in Supplementary Figure S4A and S4C.
